# Bypassing Formation of Oxide Intermediate via Chemical Vapor Deposition for the Synthesis of an Mn-N-C Catalyst with Improved ORR Activity

**DOI:** 10.1021/acscatal.3c01982

**Published:** 2023-11-01

**Authors:** Thomas Stracensky, Li Jiao, Qiang Sun, Ershuai Liu, Fan Yang, Sichen Zhong, David A. Cullen, Deborah J. Myers, A. Jeremy Kropf, Qingying Jia, Sanjeev Mukerjee, Hui Xu

**Affiliations:** † Department of Chemistry and Chemical Biology, Northeastern University, Boston, Massachusetts 02115, United States; ‡ Department of Chemical Engineering, Northeastern University, Boston, Massachusetts 02115, United States; § Giner, Inc, Newton, Massachusetts 02466, United States; ∥ Center for Nanophase Materials Sciences, Oak Ridge National Laboratory, Oak Ridge, Tennessee 37831, United States; ⊥ Chemical Sciences and Engineering Division, Argonne National Laboratory, Lemont, Illinois 60439, United States

**Keywords:** electrocatalysis, oxygen reduction reaction, PGM-free catalysts, proton exchange membrane fuel cells, in situ X-ray absorption spectroscopy

## Abstract

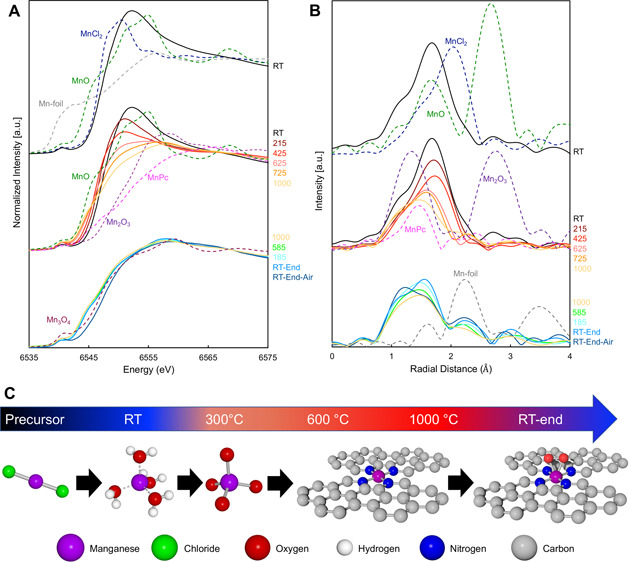

A significant barrier to the commercialization of proton exchange membrane fuel cells (PEMFCs) is the high cost of the platinum-based oxygen reduction reaction (ORR) cathode electrocatalysts. One viable solution is to replace platinum with a platinum-group metal (PGM) free catalyst with comparable activity and durability. However, PGM-free catalyst development is burdened by a lack of understanding of the active site formation mechanism during the requisite high-temperature synthesis step, thus making rational catalyst design challenging. Herein we demonstrate in-temperature X-ray absorption spectroscopy (XAS) to unravel the mechanism of site evolution during pyrolysis for a manganese-based catalyst. We show the transformation from an initial state of manganese oxides (MnO_*x*_) at room temperature, to the emergence of manganese-nitrogen (MnN_4_) site beginning at 750 °C, with its continued evolution up to the maximum temperature of 1000 °C. The competition between the MnO_*x*_ and MnN_4_ is identified as the primary factor governing the formation of MnN_4_ sites during pyrolysis. This knowledge led us to use a chemical vapor deposition (CVD) method to produce MnN_4_ sites to bypass the evolution route involving the MnO_*x*_ intermediates. The Mn-N-C catalyst synthesized via CVD shows improved ORR activity over the Mn-N-C synthesized via traditional synthesis by the pyrolysis of a mixture of Mn, N, and C precursors.

## Introduction

The promise of proton exchange membrane fuel cells (PEMFCs) as a replacement for the low-efficiency fossil fuel-burning internal combustion engines (ICEs) is hampered by the sluggish kinetics of the cathodic oxygen reduction reaction (ORR).^1,2^ These sluggish kinetics and the corrosive environment of the PEMFC cathode mandate the use of platinum group metals (PGMs) or their alloys resulting in comparatively high costs for PEMFC-based power systems versus the incumbent ICEs.^3,4^ Thus, there has been increasing effort in the development of high-performance, inexpensive ORR catalysts with the requisite activity and durability in the highly oxidizing and acidic environment of the PEMFC cathode. Currently, catalysts composed of single transition metal atoms embedded in a nitrogen-carbon matrix (TM-N-C where TM = Mn, Fe, or Co) formed through pyrolysis of TM, N, and C precursor compounds show the highest ORR activity in acidic media among PGM-free catalysts.^5−7^ In particular, the ORR activity of state-of-the-art Fe-N-C catalysts approaches that of Pt in PEMFCs.^8,9^ However, the durability of Fe-N-C needs to be significantly improved for viable applications.^10^ While improvements in recovery procedures have the potential to increase the lifetime of the Fe-N-C electrode, the fundamental lack of stability remains a primary concern.^11^ One proposed mechanism of the performance decay of Fe-N-C catalysts is the possible formation of radicals by the Fenton reaction between Fe ions and peroxide in the acidic PEMFC environment.^12−14^ Alternative transition metals such as Mn and Co have been investigated to increase the stability of these single-atom sites.^7,15^ First-principle density functional theory (DFT) calculations have shown the Co-N_4_ sites and Mn-N_4_ sites have higher intrinsic resistance to acid leaching of the metal center over their Fe counterpart, suggesting enhanced stability of these active site.^16,17^ This hypothesis led us to develop Mn-N-C catalysts.

The ORR activities of Mn-N-C catalysts reported hitherto still lag far behind those of the state-of-the-art Fe-N-C catalysts,^18−22^ thus making improved durability less meaningful. Mn-N-C catalysts often suffer from lower metal loading than their Fe and Co counterparts and require multiple doping steps or extensive post-treatment to achieve competitive activity, making the large-scale production of such materials challenging. Synthesis of the MnN_4_ site is particularly difficult owing to the propensity of Mn to form oxides at various stages of the synthesis process, limiting the amount of single-atom MnN_4_ in the catalyst. These issues drove us to improve the ORR activity of Mn-N-C catalysts by understanding the formation mechanism of Mn-N_4_ sites during pyrolysis with the aim of improving the existing synthesis methods or developing new ones. There has been much investigation into the site formation and ORR mechanisms of the Fe-N-C and Co-N-C catalysts, but much less is known for the Mn-N-C catalysts.^10,23−25^ In this work, we first studied the evolution pathway of the Mn-N_4_ site during pyrolysis of the mixture of Mn, N, and C precursors, which is also the traditional synthesis route for TM-N-C catalysts, by using *in-temperature* X-ray absorption spectroscopy (XAS). This technique has previously been used to elucidate the formation pathway of Fe-N_4_ and Co-N_4_ sites^10,26^ but not for Mn-N_4_ sites. We found that the Mn-N_4_ sites undergo a similar evolution pathway to the Fe-N_4_ and Co-N_4_ sites, that is, Mn precursors are converted to MnO_*x*_ and then to Mn-N_4_ with increasing pyrolysis temperature. However, the transition temperature from MnO_*x*_ to Mn-N_4_ is much higher than those for Fe-N_4_ and Co-N_4_ sites, hampering the selective formation of Mn-N_4_ sites. This finding led to the implementation of a novel Mn-N-C catalyst synthesized via a chemical vapor deposition (CVD) method^18^ to form Mn-N_4_ sites via trans-metalation without involving MnO_*x*_ intermediates, thereby suppressing the formation of MnO_*x*_ sites. The Mn-N-C catalyst synthesized via CVD (denoted as MnNC-CVD-T hereafter, where T is the pyrolysis temperature) exhibits a much higher ORR activity than the counterpart Mn-N-C catalyst synthesized via the traditional method of pyrolyzing a mixture of Mn, N, and C precursors. The MnNC-CVD-1100 also exhibits enhanced ORR performance and durability in PEMFCs. The critical implications of the competition between MnO_*x*_ and Mn-N_4_ during the pyrolysis are also discussed.

## Results and Discussion

### Mn-N_4_ Site Evolution

In-temperature XAS was used to unravel the enigmatic process of Mn-N_4_ site formation and evolution by designing a model system consisting of Mn, N, and C precursors capable of forming Mn-N_4_ sites. The system utilized herein was based on a metal-organic framework (MOF) ZIF-8 catalyst precursor, which has typically been used for the synthesis of TM-N_4_ single-atom sites.^6,7,10,15,27−30^ In brief, ZIF-8 with a particle size of ∼200 nm was synthesized and pyrolyzed at 1050 °C under an inert atmosphere to give highly porous nitrogen-doped carbon (NC). Subsequent ball milling of the resultant NC with anhydrous MnCl_2_ powder resulted in a mixture containing 1% Mn by weight. The mixture was then subjected to in-temperature XAS measurements under an inert gaseous environment, where the temperature was gradually raised from room temperature up to 1000 °C (upper limit of the in-temperature XAS tube furnace). The mixture was then cooled to room temperature while still under an inert atmosphere before finally being exposed to air (see the Materials and Methods in Supporting Information for further details).

**Figure 1:**
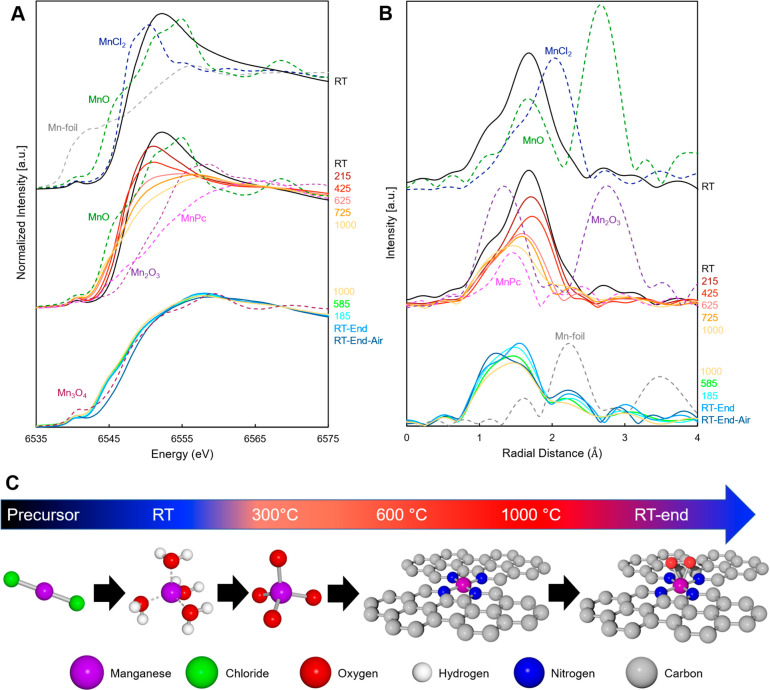
(A) XANES and (B) Fourier transform (FT)-EXAFS of MnCl_2_-NC-T collected from room temperature up to 1000 °C and while cooling back down to room temperature and subsequent exposure to air. Relevant standards collected at RT are shown as dashed lines. (C) Schematic of the proposed mechanism of active site formation from MnCl_2_ to a representative model of the final oxidized Mn-N_4_ structure derived from in-temperature XAS.

The near-edge regions of the Mn K-edge X-ray absorption (XANES) spectra and the Fourier transforms of the extended regions of the spectra (EXAFS) are shown in Figure 1A and Figure 1B, respectively, along with Mn standard compounds. A comparison of the edge energy of the experimental XANES data to that of the standards provides an indication of the predominant oxidation state of the Mn during the pyrolysis. The XANES edge energy of the ball milled mixture of MnCl_2_ and NC (denoted as MnNC-BM-RT) nearly overlaps that of the anhydrous MnCl_2_ precursor, indicating that the Mn in the mixture retains the +2 oxidation state of MnCl_2_ (Figure 1A). Contrarily, the Fourier transform of the extended X-ray absorption fine structure (EXAFS) for MnNC-BM-RT shows that the first shell scattering peak at ∼1.7 Å no longer matches that of the Mn–Cl scattering path observed in MnCl_2_ (∼2.0 Å) but matches the Mn–O path length of the MnO standard (Figure 1B). The fitting result quantitatively shows that the Mn–O bonds have a coordination number of 3.9 ± 0.7 with an average bond distance of 2.17 ± 0.02 Å (Table S1). This rather long bond distance is comparable to that of an Mn aquo complex ([Mn(H_2_O)_6_]^2+^) or MnCl_2_·4H_2_O.^31^ Similar to these compounds, the MnNC-BM-RT does not have the Mn–Mn scattering peak that exists in Mn oxides such as MnO (Figure 1B), indicating the lack of a long-range-ordered structure of Mn and that Mn is present as atomically dispersed atoms throughout the precursor. These results indicate that the MnCl_2_ was converted into the Mn-(H_2_O)_4_ complex or hydrated MnO_*x*_ with the chloride ligands being removed during ball milling. The ball milling was carried out under air with residual moisture providing the driving force for this conversion, as there is a high propensity for MnCl_2_ to form the hydrate.

During the increasing temperature profile, when the temperature was increased stepwise from RT to 425 °C, there was a marked shift in the XANES edge to lower energy, approaching the XANES spectrum of MnO, accompanied by a decrease in the white line intensity (Figure 1A). Concomitantly, there was an increase in the pre-edge peak intensity around 6540 eV as evident in the first derivative of the XANES spectra (Figure S1), signifying an increase in the local disorder. The absence of the shoulder around 6544 eV, which is present in the first derivative XANES spectrum of the manganese phthalocyanine (MnPc) as a fingerprint of the square-planar Mn-N_4_ structure, indicates that the Mn(II)-N_4_ moiety was not yet formed at 425 °C. The concurrent EXAFS spectra show a shift in the first shell scattering peak to a lower radial distance, signifying the shortening of Mn–O bonds. Meanwhile, the corresponding XANES spectra shift signifies a decrease of the first-shell Mn–O bond length and increase in local disorder, indicative of the transformation from single-atom Mn-(H_2_O)_4_ to single-atom tetrahedral Mn(II)-O_4_ with the temperature increase from RT to 425 °C. This observation is similar to our previous observation of the conversion from Fe oxides to single-atom tetrahedral Fe(II)-O_4_ via a crystal-to-melt-like transformation as the temperature increased from RT to 600 °C.^10^

A shoulder around 6544 eV becomes discernible in the first derivative XANES spectrum as the temperature was increased to 725 °C, providing evidence of the formation of square-planar Mn(II)-N_4_ (Figure S1). This process is analogous to that of iron, albeit at a temperature 100 °C higher than that observed for Fe. The intensity of this shoulder continues to increase as the temperature progresses toward 1000 °C, confirming the continuous transformation from Mn(II)-O_4_ to Mn(II)-N_4_. This transformation is further supported by the XANES and Fourier transform of the extended X-ray absorption fine structure (FT-EXAFS) in the form of the overall shape of XANES spectra approaching that of MnPc with increasing temperature. Simultaneously, the FT-EXAFS peak around 1.7 Å shifts negatively to a smaller radial distance, indicating the shortening of the first-shell bond length as expected for the transformation from tetrahedral Mn-O_4_ to square-planar Mn(II)-N_4_. EXAFS fitting confirms that the bulk-average first-shell bond distance decreases to ∼2.00 Å (Table S1). This bond distance is, however, still longer than that of MnPc (1.96 Å). We postulate that this bond distance is the bulk-average result of the Mn–N bond distances representing a combination of the shorter Mn–N bond in the Mn-N_4_ sites and the relatively long Mn–O bonds from the Mn–O_4_, and it could also be a result of Mn being slightly out of the N_4_ plane. It should be noted that the Mn–N and Mn–O scattering coincides in the EXAFS data and thus N and O cannot be distinguished using either FT-EXAFS or EXAFS fitting alone. However, the breadth of the FT-EXAFS peak is indicative of the copresence of multiple bonds (Mn–N and Mn–O) with similar bond lengths (Figure 1B). Additionally, the overall shape of the XANES spectrum, even at 1000 °C, is still far away from that of MnPc, and the square-planar fingerprint shoulder is not discernible (Figure 1A), which indicates that a significant amount of Mn-O_4_ moieties still exist at 1000 °C and therefore an incomplete transformation from tetrahedral Mn-O_4_ to square-planar Mn-(II)N_4_, in agreement with the interpretation of the EXAFS data. These results differ from the iron^10^ and cobalt^26^ cases for which the Fe(II)-N_4_ and Co(II)-N_4_ moieties are the dominant species at 1000 °C. The difference suggests that a higher temperature is required to convert Mn-O_4_ to Mn-N_4_ than Fe-O_4_ and Co-O_4_ to Fe-N_4_ and Co-N_4_, respectively.

The XANES and FT-EXAFS spectra undergo little change during the cooling process other than an increase in the FT-EXAFS peak intensity, attributed to a decrease in the temperature-dependent Debye–Waller factor. The Debye–Waller factor is a measure of scattering caused by thermal motion, which increases with increasing temperature, resulting in the dampening of the EXAFS oscillations.^32^ These results show that the oxidation state was maintained, and the in-plane Mn(II)-N_4_ structure formed at higher temperatures was preserved even at lower temperatures as long as the atmosphere around the catalyst was O_2_-free. However, after the sample was exposed to air, the XANES spectrum underwent a significant positive shift toward higher energy, even surpassing that of the Mn_3_O_4_ standard (Figure 1A). Concurrently, the primary FT-EXAFS peak shifts negatively, reflecting the shortening of the bond lengths (Figure 1B). These results together suggest that both the Mn(II)-N_4_ and Mn(II)-O_4_ sites are partially oxidized by the O_2_ in air, thus forming O_2_-Mn(III)-N_4_ and Mn(III) oxides, respectively. Oxidation of the Mn(II)-N_4_ site by O_2_ is an essential first step for the initiation of the ORR mechanism and demonstrates the importance of the oxidation state change on the ability of the Mn-N-C catalysts for the ORR. However, the possibility that the Mn(II)-N_4_ sites transform back to Mn(III) oxides exposed to air cannot be excluded. Shortening of the bond length could indicate formation of an additional ligand in the form of O_2_-Mn(III)-N_4_ and some Mn(III) oxides; however, a definite assignment of a structure is difficult due to difficulty in deconvolution of the nearest neighbors of the O and N by EXAFS fitting.

In principle, the site evolution of the Mn pathway is analogous to that of Fe and Co identified previously.^10,26^ That is, the precursor transforms to oxides below 500–600 °C, and the oxides then transform to TM(II)-N_4_ moieties at higher temperatures. These results together suggest a universal evolution pathway of TM(II)-N_4_ sites in the traditional synthesis method that pyrolyzes the mixture of TM (TM= Mn, Co, Fe), N, and C precursors: precursor → oxide → TM(II)-N_4_. This pathway was recently extended to 37 elements, including Mn, Fe, and Co, using in-temperature XAS as well, together with other techniques.^33^ However, the Mn pathway differs from that of Fe and Co in the temperature of the phase transition. The threshold temperature for the formation of Mn(II)-N_4_ (725 °C as defined by the emergence of the square-planar fingerprint peak in the first derivative of the XANES spectrum) is higher than that of Fe(II)-N_4_ (600 °C) and Co(II)-N_4_ (∼523 °C). In addition, the transformation from oxides to TM(II)-N_4_ is nearly complete at 1000 °C for Fe and Co but far from complete for Mn. These results together show that there is a competition between TM oxides (such as tetrahedral TM-O_4_) and planar-square TM(II)-N_4_ sites, and the high-temperature pyrolysis in an O_2_-free environment drives the transformation from TM oxides to TM(II)-N_4_ sites. An inherent factor that determines the degree of competition is the relative thermodynamic stability between the TM oxides and TM(II)-N_4_. Specifically, the TM with higher oxophilicity (or equivalently strong TM-O binding energy) requires a higher pyrolysis temperature to form TM(II)-N_4_ sites. This is supported by the trend of the threshold temperature for the formation of TM(II)-N_4_ sites: Mn > Fe > Co. The high pyrolysis temperature of Mn(II)-N_4_ is disadvantageous since it destabilizes nitrogen species and graphitizes carbon, especially in the presence of TM, therefore limiting the TM(II)-N_4_ site density.^28^ This new understanding drove us to seek an alternative route for the synthesis of Mn-N-C catalysts that bypass the formation of MnO_*x*_.

#### Synthesis and Characterization of MnNC-CVD Catalyst

**Figure 2:**
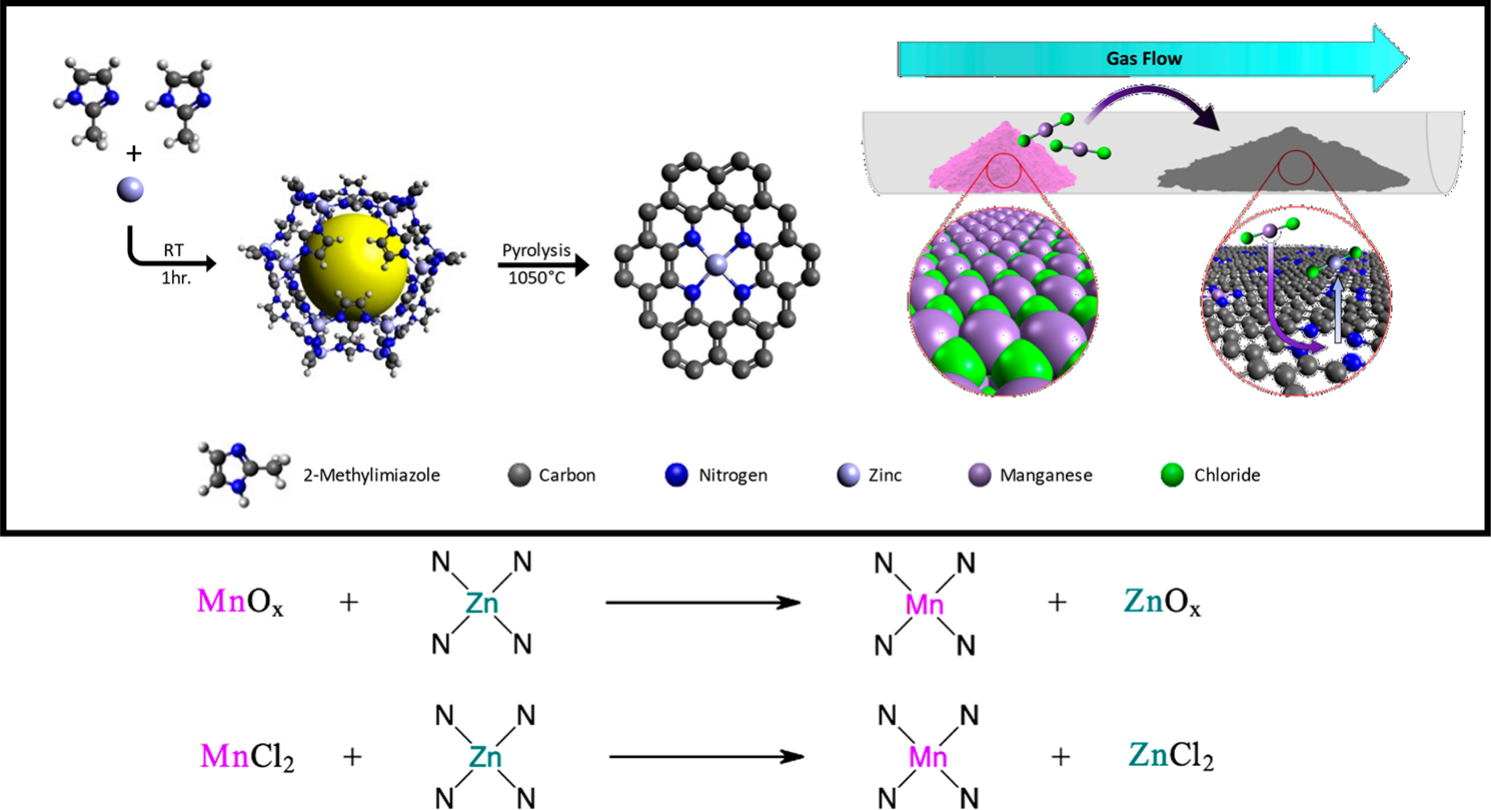
Formation of the MnN_4_ active sites. (A) Synthesis scheme of Mn-N-C, from ZIF-8 synthesis to formation of the nitrogen-doped carbon via pyrolysis, then to chemical vapor deposition (CVD) forming the MnN_4_ site. (B) Proposed mechanism of incorporation of manganese into the nitrogen-doped carbon by the traditional method of ball milling/heating and (C) via the chemical vapor deposition method.

We envisaged that the chemical vapor deposition (CVD) method that we previously developed for the synthesis of Fe-N-C catalysts might be a more suitable method to synthesize Mn-N-C (Figure 2A) since it enables direct transformation from iron chloride to Fe-N_4_ sites via transmetalation, without going through iron oxides (Figure 2B). The transmetalation mechanism enabled by CVD allows for the formation of Fe-N_4_ directly from the FeCl_3_ precursor rather than going through Fe oxides, which lowers the optimal pyrolysis temperature by ∼200 °C. We, therefore, implemented the CVD method to synthesize Mn-N-C catalysts to presumably form the Mn-N_4_ sites directly from Mn precursors governed by the same transmetalation mechanism (Figure 2C). The preferred precursors for CVD are TMCl_*x*_ compounds due to the formation of ZnCl_2_ as a product (Figure 2C). ZnCl_2_ has a lower boiling point (732 °C) than Zn metal (907 °C) which is also lower than the temperature for the carbothermic reduction of ZnO (950 °C), thus facilitating the removal of Zn.^18,34^ An additional benefit of the CVD process is that the transformation occurs at gas phase accessible sites, resulting in surface-decorated catalyst sites that are available for the ORR.

**Figure 3:**
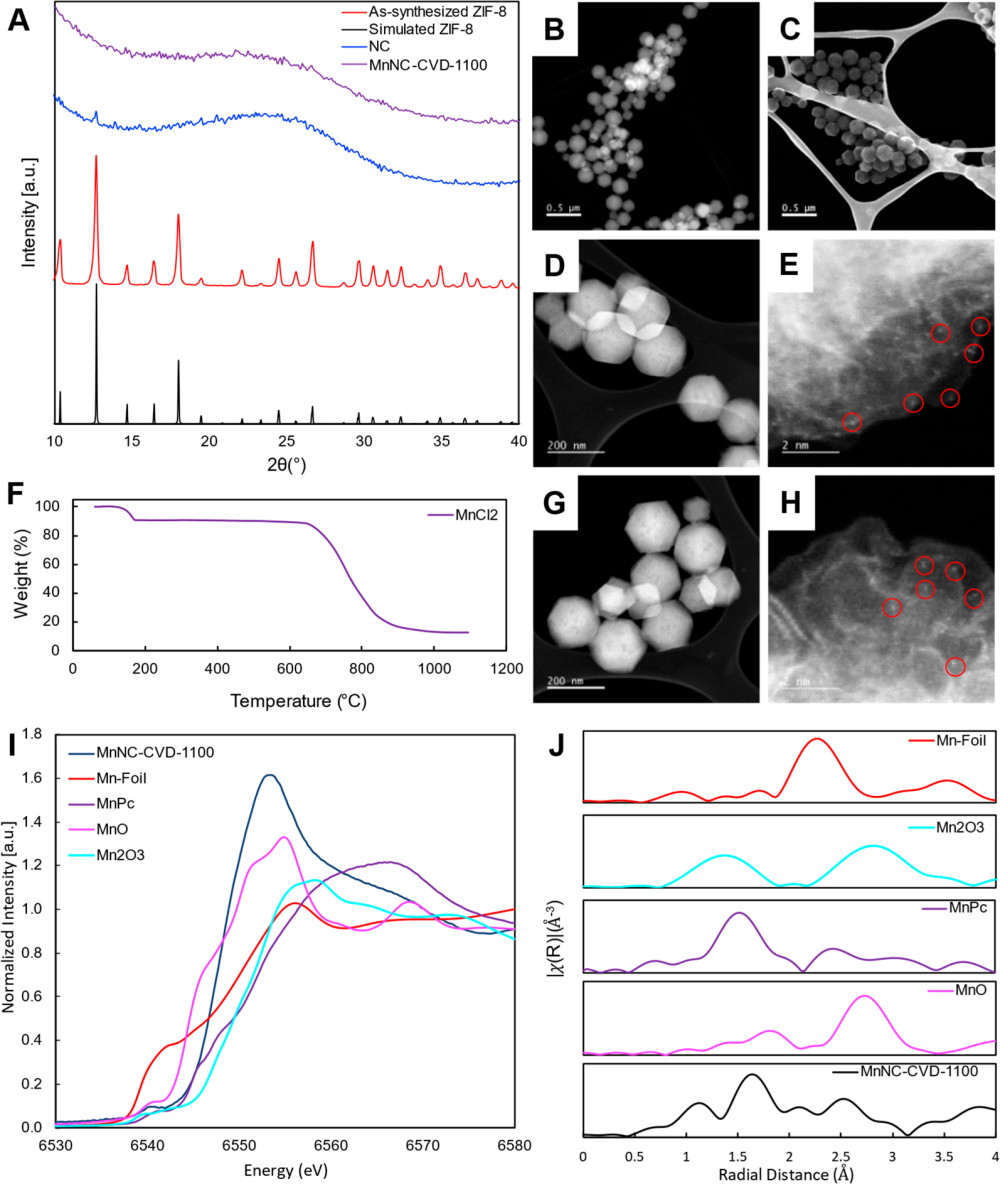
Physical characterization of the MnNC-CVD-1100 catalyst and its precursors. (A) X-ray diffraction patterns of the MnNC-CVD-1100 catalyst, the nitrogen-doped carbon, the as-synthesized ZIF-8, and a simulated ZIF-8 pattern. (B,C) STEM images of the ZIF-8 particles. (D,E) STEM images of the nitrogen-doped carbon with selected zinc atoms circled in red. (F) Dynamic thermogravimetric analysis of the anhydrous MnCl_2_ under Ar atmosphere. (G,H) STEM images of the MnNC-CVD-1100 with selected Mn atoms circled in red. (I) Ex situ Mn K-edge XANES of MnNC-CVD-1100 and relevant Mn standards. (J) Ex-situ Mn K-edge FT-EXAFS of MnNC-CVD-1100 and relevant Mn standards.

The first step of the CVD method is the same as the traditional approach in that it requires a porous nitrogen-rich carbon matrix with a plethora of ZnN_4_ sites available for conversion into the MnN_4_ sites. This was achieved by using ZIF-8 as a template, which was synthesized via the methanol solvent-based approach that produced ZIF-8 as a highly ordered crystal structure, as measured using X-ray diffraction (XRD). This result matches well with the pattern simulated from the crystal structure, as shown in Figure 3A.^35^ The ZIF-8 had an average particle size of 150–230 nm, confirmed by aberration-corrected scanning electron microscopy (AC-STEM) (Figure 3 B,C). This material underwent pyrolysis at 1050 °C under inert conditions to convert it into a highly porous carbon matrix structure which contained 5.19 wt % N and 2.49 wt % Zn (Table S2). After pyrolysis, the particle shape was maintained, but the size was reduced to ∼150 nm, as seen using AC-STEM (Figure 3D). Annular dark-field (ADF) STEM reveals the presence of metal atoms as bright spots throughout the pyrolyzed ZIF-8 particles (Figure 3E). When electron energy loss spectroscopy (EELS) was performed on these areas, the expected Zn L-edge was not observed and only the C and N peaks were detected (Figure S3). From the elemental analysis of the sample, the predominant metal was zinc, which indicates these bright spots are likely zinc sites. The lack of the Zn L-edge in the EELS is attributed to the high mobility of the Zn atoms, which causes the Zn to leave the sites when exposed to the high-energy electron beam during analysis. XRD demonstrates that highly crystalline ZIF-8 was converted into an amorphous and graphitic matrix, as seen from the powder diffraction pattern (Figure 3A), with no noticeable crystalline phases. MnCl_2_ was chosen as the Mn precursor as it would form volatile ZnCl_2_ during the transmetalation process and the vaporization temperature of the Mn is below that used for the CVD process, as seen from the thermogravimetric analysis of anhydrous MnCl_2_, with vaporization starting at 750 °C (Figure 3F). The CVD process was subsequently employed where MnCl_2_ and NC were heated to 1100 °C in an inert gas flow in a tube furnace, with MnCl_2_ upstream of the NC, to produce the MnNC-CVD-1100 catalyst. In this arrangement, heating of MnCl_2_ caused its vaporization and transport to the NC via the inert gas stream where it deposited on the surface of the NC and was eventually converted to MnN_4_ via the transmetalation process, replacing Zn in the ZnN_4_ sites. This process completely removed Zn from the catalyst, and Mn was incorporated into the matrix at a concentration of 1.01 wt %. The high pyrolysis temperatures required for the conversion to MnN_4_ resulted in a decrease of nitrogen from 5.19 wt % to 2.68 wt %, which dramatically lowers the upper limit of the theoretical site density. AC-STEM imaging of the MnNC-CVD-1100 catalyst shows that it retains the structure and particle size during the CVD process (Figure 3G). The bright dots in the ADF-STEM image of the MnNC-CVD-1100 catalyst (Figure 3H) were investigated via EELS and showed Mn atoms surrounded by nitrogen (Figure S4). Ex situ XAS was performed on the MnNC-CVD-1100 catalyst. The XANES edge shift was similar to that of the ball milled in-temperature XAS sample after exposure to air but with an increase in the white line intensity (Figure 3I). The disappearance of the shoulder in the XANES derivative spectra at 6544 eV is also consistent with the ball milled in-temperature XAS sample after exposure to air (Figure S5), suggesting the oxidation of MnN_4_ sites upon exposure to air. The atomic dispersion of Mn throughout the catalyst was confirmed by the lack of observed crystallinity in the XRD pattern (Figure 3A), the absence of long-range scattering in EXAFS (Figure 3J), and the observed spacing of the Mn atoms in the ADF-STEM measurements (Figure 3H). The CVD approach represents a clear pathway *sans* oxide intermediate, with the resultant catalyst expected to have a lower oxide content as compared to the ball milled and pyrolyzed samples.

#### Electrochemical Characterization of MnNC-CVD

**Figure 4:**
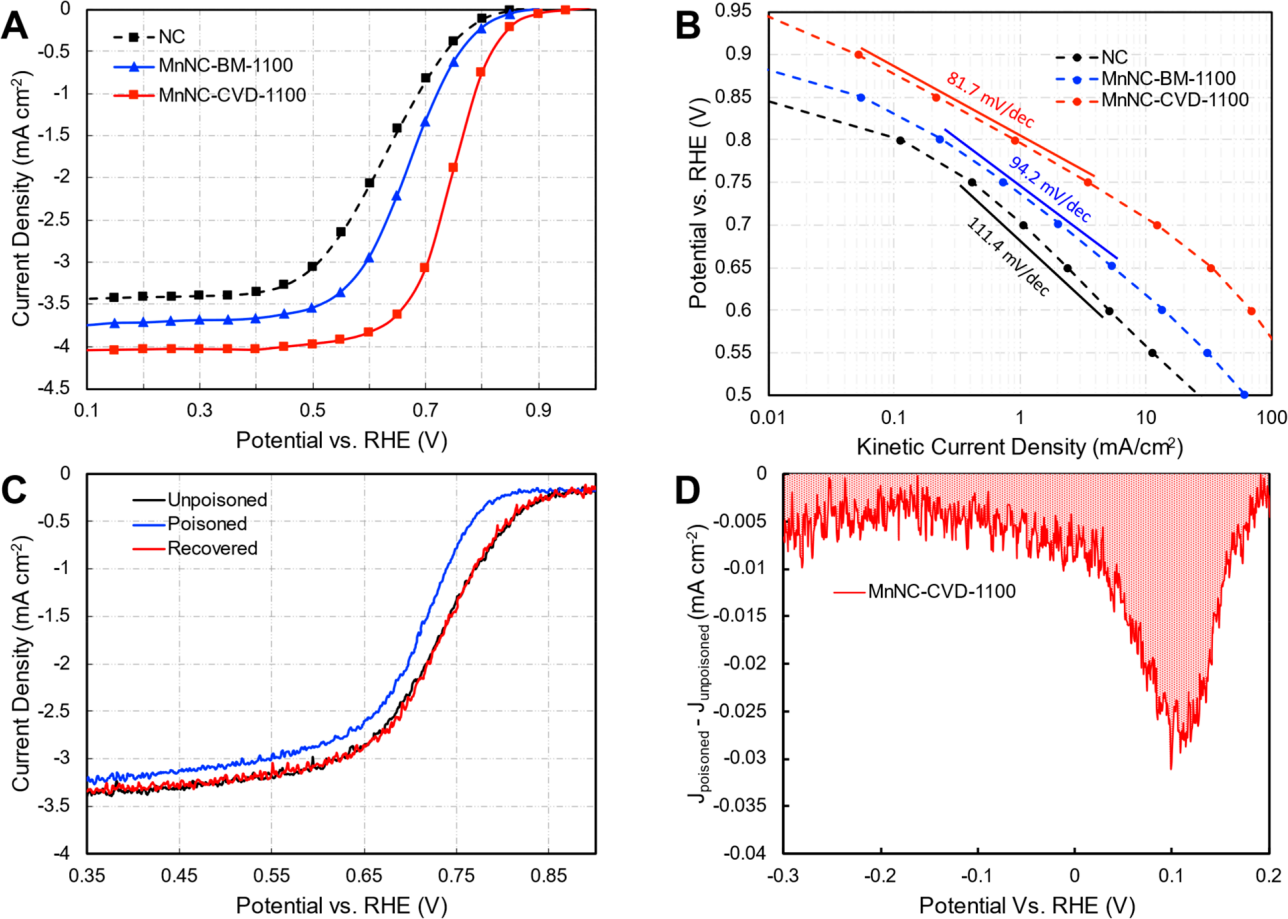
Electrochemical ORR Performance of MnNC-CVD-1100. (A) Steady state rotating disk electrode (RDE) ORR Polarization curves were obtained in an O_2_-saturated 0.5 M H_2_SO_4_ electrolyte at room temperature, 900 rpm, and a loading of 0.8 mg cm^–2^. All potentials are versus the reversible hydrogen electrode (RHE). (B) The Tafel slopes were obtained from the RDE ORR polarization curve in A. (C) ORR activity of the MnNC-CVD-1100 catalyst initially poisoned with NO_2_, and recovered after stripping; in O_2_-saturated 0.5 M acetate buffer (pH 5.2) 0.27 mg cm^–2^ loading and a rotation rate of 900 rpm and scanned negatively at 5 mV/s. (D) NO_2_ stripping current from 0.2 to −0.3 V vs RHE with a scan rate of 10 mV s^–1^.

The ORR activity of the catalyst synthesized by different methods, i.e. chemical vapor deposition (MnNC-CVD) and ball milling (MnNC-BM), were first evaluated using the rotating disk electrode (RDE) technique (Figure 4A). The catalyst synthesized via CVD, MnNC-CVD-1100, compared to the ball milled, MnNC-BM-1100, at the same temperature of 1100 °C, shows a significant increase in ORR activity which indicates that the CVD method resulted in higher active site density than the traditional method. This is due partly to the suppression of the MnO_*x*_ by bypassing the Mn(II)-O_4_ intermediate through the transmetalation with ZnN_4_.

Quantitative analysis using Levich equation of the RDE data provided a relative comparison of the number of electrons transferred during ORR, wherein the MnNC-CVD-1100C (Figure 4C) achieves an electron transfer number of 3.94 compared to corresponding value for MnNC-BM-1100 of 3.64 (equation S1), demonstrating the selectivity of the CVD catalyst for promoting the four-electron reduction of O_2_ to water rather than the two-electron reduction to peroxide. Loading studies conducted on the MnNC-CVD-1100 catalyst show similar limiting currents with lower loadings down to 0.4 mg cm^–2^ (Figure S6). This indicates a higher likelihood of a direct four electron transfer mechanism rather than the 2 + 2 electron mechanism via a peroxide intermediate. The latter would be more susceptible to a loading dependence of limiting current density.^36^ Though detailed Tafel analysis of the ORR reaction ideally should utilize a polished metal surface, as per the original Levich formulation of the technique, analysis of the Tafel slopes on dispersed electrodes such as those used here provide information about the rate limiting steps. The theoretical value of the Tafel slope of 120 mV dec^–1^ indicates that the first charge transfer step of M-OOH to M-OOH^–^ is limiting and the lower Tafel slope of 60 mV dec^–1^ can be attributed to a protonation and coupled charge transfer of adsorbed oxygen (M-OO) to give M-OOH as such the Tafel slope of 81.7 mV dec^–1^ exhibited by MnNC-CVD-1100 (Figure 4B) suggests a mixed rate-limiting step.^37^

Active site density calculations were conducted via NO stripping. The shift of the ORR half-wave potential to lower potentials after the exposure of the catalyst to NO_2_^–^ to form adsorbed NO indicates that active sites were blocked by this probe molecule (Figure 4C). The charge for stripping of the NO from the active sites was determined to be 1.45 C g^–1^, calculated from the area of the difference in the stripping current of the poisoned vs the pristine, unpoisoned, catalyst (Figure 4D). From equation S3, the catalyst surface area was calculated to be 726 m^2^ g^–1^ based on the capacitance of the MnNC-CVD catalyst (Figure S7) in the cyclic voltammograms acquired in deaerated electrolyte. The site density can be calculated via equation S4. The active site density of this catalyst using NO stripping is 2.9 × 10^15^ site m^–2^. Using this site density and the current from the ORR RDE traces, the ORR turnover frequency was calculated to be 3.1 e^–^ site^–1^ at 0.8 V vs RHE. It should be noted that the site density was calculated with the assumption of a 5-electron reduction of the adsorbed NO to ammonia (equation S2); however, it has been suggested that a 3-electron reduction process may be dominant, meaning the actual site density may be higher.^38^ To this point, the characterization of the site density of PGM-free sites is highly dependent on the method, thus making it difficult to obtain an accurate evaluation of site density. This can lead to an underestimation of the site density and an overestimation of turnover frequency (TOF) when compared to other TMN_4_ catalysts (Table S3). It has been seen that NO from NO_2_ can be absorbed onto Fe oxide sites; this may not be the case for Mn oxides, causing a discrepancy in the quantification of MnN_4_ active sites as compared to FeN_4_ sites.^13^

**Figure 5:**
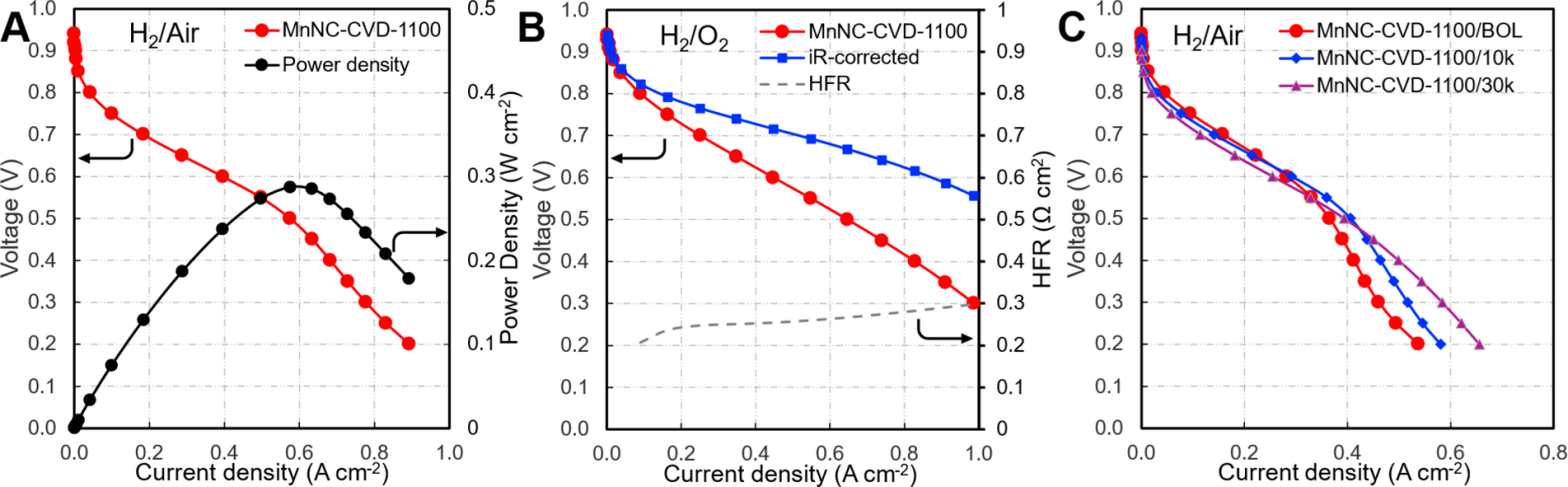
(A) PEMFC polarization curve and power density of the MnNC-CVD-1100 under H_2_/Air conditions 4 mg_cat_ cm^–2^ cathode and 0.3 mg_Pt_ cm^–2^ anode loadings with a 5 cm^2^ electrode area, Nafion 212 membrane and 80% relative humidity at 80 °C with 1000 mL min^–1^ air/O_2_ and 200 mL min^–1^ H_2_ flow, and 1.0 bar back pressure of reactant gas. (B) PEMFC performance under H_2_/O_2_ conditions, 6 mg_cat_ cm^–2^ cathode loading. Also shown are the *iR*-corrected polarization curve and the high-frequency resistance used to calculate the *iR* correction. (C) ORR stability of the catalyst in relation to potential cycling under nitrogen. All curves were not *iR*-corrected, unless otherwise noted.

In addition to the kinetic data at the RDE level, the MnNC-CVD-1100 catalyst was tested in membrane-electrode assemblies. The activity of the catalyst, represented by the current density at a 0.9 *iR*-corrected voltage under H_2_/O_2_, is 8.7 mA cm^–2^ (Figure 5B). The cell also demonstrates reasonable H_2_/Air performance, a current density of 189 mA cm^–2^ at 0.7 V, and a maximum power density of 287 mW cm^–2^ at 0.6 V making it comparable to the performance of other manganese-based PGM-free catalysts (Figure 5A).^7,39^ However, H_2_/Air performance is still much lower than that of the state-of-the-art Fe-N-C catalysts. A Fe-N-C catalysts synthesized from ZIF-8 and iron oxide and subsequently treated with ammonium chloride salt was able achieve power a densities of 601 mW cm^–2^ under H_2_/Air conditions, suggesting the need for increased activity of the Mn based catalysts.^40^ The catalyst stability in the PEMFC was tested via an accelerated stress test where the cell was cycled between 0.6 and 1.0 V at 50 mV s^–1^ under H_2_/N_2_ at 80 °C and 80% relative humidity. After 30k cycles of the accelerated stress test, there was a 34 mV decrease in cell voltage at a current density of 0.44 mA cm^–2^, indicating decent stability toward of the catalyst, just missing the DOE target of <30 mV (Figure 5C). Comparing the voltage at 0.8 V, where the current is primarily limited by ORR kinetics and not mass transport, the current decreased by 34% after 10,000 cycles. This is similar to the case for Fe based catalyst made using the traditional ball milling method, which decreased by 39% at 0.8 V.^41^ This is consistent with previous comparisons where Fe-N-C catalysts exhibit a much faster initial degradation rate, more than twice that of the Mn catalysts in the first 20 h. The rapid degradation in the initial phase can be mainly attributed to the metal leaching caused by bond breaking between the metal center and adjacent nitrogen atoms. The much faster degradation of the Fe-N-C catalyst is associated with the fragile nature of the Fe–N bond, as elucidated by using DFT studies.^39^ Thus, the new Mn-N-C catalyst could be more durable than Fe-N-C catalysts because of the robust nature of the MnN_4_ sites as well as enhanced resistance to carbon corrosion. By comparing the carbon structure of those Mn-N-C and Fe-N-C catalysts, a more predominantly graphitic carbon structure was observed for the Mn-N-C catalyst, as reported earlier. The mass transport region of the *I*–*V* curve was greatly improved upon cycling, which may be attributed to the pores opening during voltage cycling and the subsequent mitigation of water flooding. However, it should be noted that PGM-free catalysts are much more susceptible to degradation when cycling in O_2_ rather than N_2_.^39,42^

Further work to increase the Mn-N-C catalysts will be needed in terms of both durability and stability. Currently, the intrinsic tendency of Mn to form oxides can hinder both of these aspects. Limited Mn site formation and degradation of these under PEMFC operating conditions is a significant concern.^30^ Controlling synthesis conditions have been shown to be effective in increasing activity by increasing the nitrogen content of the base N-doped carbon to allow for higher site density. Additionally, the stability of the nitrogen within the carbon structure is important, as high temperatures are required to drive the synthesis of the MnN_4_ active site. Increasing the stability of the Mn-N-C under operating conditions must be considered for particle applications of such catalysts; however, many current techniques come at the cost of activity, which Mn-N-C-based catalysts cannot afford to lose. Shielding the active sites with carbon has been demonstrated to increase the stability of M-N-C’s but often reduces the performance of the catalyst.^43^ Focus on strengthening the Mn–N bond by optimizing the pyridinic to pyrrolic nitrogen ratio seems to be the most promising approach to increase durability while still leaving the sites available for the ORR.

## Conclusions

In conclusion, the formation of the MnN_4_ site was found to be dependent on the ability of the synthetic method to suppress MnO_*x*_ formation and to promote MnN_4_ production. This can be facilitated by increasing pyrolysis temperatures to drive the reaction toward the formation of MnN_4_ sites or by the CVD synthesis method which eliminates the MnO_*x*_ intermediate step in the pyrolysis process. However, the high pyrolysis temperatures required for Mn-N_4_ formation limit the number of active sites available, as there is a consequential decrease in nitrogen content with increasing temperatures. Nevertheless, the CVD method produced a Mn-N-C catalyst with high ORR activity in RDEs and good performance and durability in PEMFCs.

## Supporting Information Available

Materials and methods, physical characterizations, electrochemical characterizations, tables and figures with additional data, supplemental reference (PDF)
