# Supplementary material for: Bypassing Formation of Oxide Intermediate via Chemical Vapor Deposition for the Synthesis of an Mn-N-C Catalyst with Improved ORR Activity
Source: ACS Catal. 2023 Nov 1;13(22):14782–91. doi: 10.1021/acscatal.3c01982 (PMC10660335; doi:10.1021/acscatal.3c01982)
Supplement: Supplementary file 1 — cs3c01982_si_001.pdf [file cs3c01982_si_001.pdf]

Supporting Information

Bypassing formation of oxide intermediate via chemical vapor deposition for the synthesis of an Mn-N-C catalyst with improved ORR activity

Thomas Stracensky[1], Li Jiao[2], Qiang Sun[1], Ershuai Liu[1], Fan Yang[3], Sichen Zhong[3], David A. Cullen[4], Deborah J. Myers[5], Qingying Jia[1], Sanjeev Mukerjee[1], Hui Xu[3]

[1]Department of Chemistry and Chemical Biology, Northeastern University, Boston, Massachusetts, 02115, United States

[2]Department of Chemical Engineering, Northeastern University, Boston, Massachusetts, 02115, United States

[3]Giner, Inc, Newton, Massachusetts, 02466, United States

[4]Center for Nanophase Materials Sciences, Oak Ridge National Laboratory, Oak Ridge, Tennessee 37831, USA

[5]Chemical Sciences and Engineering Division, Argonne National Laboratory, Lemont, Illinois, 60439, United States

Materials and Methods

Synthesis

Chemicals: Manganese chloride anhydrous ($MnCl_2$, ≥99.99%) 1,10 -phenanthroline anhydrous, Zinc nitrate hexahydrate ($ZnNO_3 \cdot 6H_2O$), 2-methylimidazole, manganese (II) phthalocyanine (MnPc), were purchased from Sigma-Aldrich. Sulfuric acid (H_2SO4, 98%, ACS grade) and methanol (ACS grade) were purchased from Fisher scientific. All aqueous solutions were prepared using deionized (DI) water (18.2 MΩ·cm) obtained from a Millipore Elix 5 purification.

Synthesis of ZIF-8: Zinc nitrate was dissolved in 200 mL of methanol to form a 0.1 M solution (Solution A), and 2-methylimidazole was dissolved in another 200 mL of methanol to form a 0.4 M solution (Solution B). Next, solution A was poured into Solution B, and the mixture was stirred at room temperature for one hour. The solution was then centrifuged at 10,000 RPM for ten minutes and the ZIF-8. The solid ZIF-8 was collected and washed four times with methanol and then dried at 60 °C in a vacuum oven overnight.

Synthesis of N-C: 100 mg of ZIF-8 was transferred into a quartz boat and placed into a tube furnace purged with argon. The sample was ramped to 1050 °C at a rate of 5 °C/min and held at 1050 °C for 1 hour before allowing it to cool down to room temperature.

Synthesis of Mn-N-C by CVD: 30 mg of previously prepared NC was placed at one end of a quartz boat, and 15 mg of anhydrous $MnCl_2$ was placed on the other end. The boat was placed in the tube furnace with the NC oriented downstream of the $MnCl_2$, and the tube was purged with argon. The sample was ramped to 1100 °C at a rate of 30 °/min and held at 1100 °C for 1 hour before allowing it to cool down to room temperature.

Synthesis of Mn-N-C catalyst for in temperature XAS: 400 mg of NC and 14.3 mg of anhydrous $MnCl_2$ were added to a container under atmosphere with five 0.25" methyl methacrylate balls. The mixture was then ball milled for 3 hours to obtain a powder with ~1% Mn content. This powder was then used for in-temperature XAS.

Physical characterizations

Scanning transmission electron microscope:

AC-STEM was conducted using a JEOL NEOARM TEM/STEM operated at 80 keV and equipped with a Gatan Quantum electron energy-loss spectrometer and dual 100 m^2 silicon drift detectors for energy-dispersive X-ray spectroscopy.

X-ray diffraction:

X-ray diffraction (XRD) patterns were obtained using a Rigaku Ultima IV x-ray diffractometer with a Cu K_α source (λ = 1.541 Angstroms) operated at 40 kV and 44 mA. The XRD patterners were analyzed using Rigaku's PDXL software. ZIF-8 powder diffraction pattern was simulated using VESTA software version 3.5.7 and a ZIF-8 single crystal structure data.[1]

Thermogravimetric analysis:

Thermogravimetric analysis (TGA) of $MnCl_2$ was done using an SDT Q600 Thermal analyzer. A small sample of ~4mg was placed into an aluminum crucible, and the furnace was purged with argon for 30 minutes. The sample was then ramped at 5 C/min to 1100C. The TGA curves were analyzed using TA Instruments' Universal Analysis 2000 software.

Elemental analysis

Elemental analysis of MnNC-CVD-1100 and NC samples were conducted by Robertson Microlit Laboratories by inductively coupled plasma atomic emission spectroscopy for Mn, Zn, and Fe, combustion analysis for H, C, and N, and ion chromatography for Cl.

In-temperature X-ray absorption spectroscopy:

The in-temperature XAS measurements during pyrolysis were performed at beamline 10-ID of the Materials Research Collaborative Access Team (MRCAT) at the Advanced Photon Source, Argonne National Laboratory, Lemont, Illinois, United States. The experimental apparatus for these experiments and the results of the first use of the apparatus to study the evolution of the Fe speciation during heat treatment of Fe-doped ZIFs are described in Myers et al.[2–5]. The XAS data were processed and fitted using the IFEFFIT−based Athena and Artemis programs.[6] Scans were calibrated, aligned, and normalized with the background removed using the IFEFFIT suite.[6] The $\chi(R)$ were modeled using single scattering paths calculated by FEFF6.[7]

Electrochemical characterization

Rotating disk electrode:

Catalyst inks for RDE and RRDE studies were made by dispersing 5mg of catalyst into 285 μL of isopropyl alcohol and 15 μL of 5 wt% Nafion solution (Ion Power) and sonicated for 30 minutes. 15 μL of catalyst dispersion was then drop-cast onto the glassy carbon electrode (0.247 cm^2, Pine instrument), resulting in a loading of 0.8 mg cm^{-2}. All the electrochemical experiments were conducted using a three-electrode cell system using a Pine instrument rotator and a Princeton Applied Research VersaSTAT 4 potentiostat. A graphite rod and a Gaskatel

HydroFlex® hydrogen reference electrode were used as the counter and reference electrodes respectively.

Proton-exchange membrane fuel cell:

The Mn-N-C catalyst was used to prepare the cathode for MEA tests in a PEMFC under H_2-O_2 conditions, followed by an H_2-air test. The cathode catalyst inks were prepared by dispersing the calculated amount of catalyst powder and Nafion D521 dispersion (Ion power) into 50 wt/% 1-propanol aqueous solution for 3 hours under ice bath sonication. The inks were coated layer by layer on an SGL 29-BC gas diffusion layer (Sigracet) until 4 mg (or 6 mg) catalyst cm^{-2} loading was achieved. A commercial Pt gas diffusion electrode (0.3 mgPt cm^{-2}, fuel cell store) was used as the anode. The anode electrode was first heat pressed onto NR212 membrane (Ion Power) at 130 °C for 4 minutes. Before hot pressing the cathode on the opposite side of the membrane, a thin Nafion overspray layer was applied on the cathode catalyst layer to reduce the interfacial resistance. The cathode was then hot pressed on the previously-pressed half MEA at 130 °C for 2 minutes. The MEA was subsequently assembled into a single cell with single -serpentine flow channels. The single cell was then evaluated in a fuel cell test station (100 W, Scribner 850e, Scribner Associates). The cells were conditioned under an N_2 environment at 100% relative humidity and at 80 °C for 2 hours. Air/oxygen flowing at 1000 ml min^{-1} and H_2 (purity 99.999%) flowing at 200 ml min^{-1} were used as the cathode and anode reactants, respectively. The back pressures during the fuel cell tests are 1.0 bar reactant gas. The vapor pressure is around 0.5 bar owing to the 100% relative humidity. Thus, the total pressure applied to the MEA is around 1.5 bar (150 KPa). Fuel cell polarization curves were recorded in a voltage control mode.

Levich current analysis:

The Levich current can give information on the number of electrons transferred during a reaction, with a 4-electron transfer indicating the complete reduction of O_2 to water and a 2-electron transfer suggesting a partial reduction to H_2O_2.

$$I_L = (0.620)nFAD^{\frac{2}{3}}\nu^{\frac{1}{6}}C\omega^{\frac{1}{2}} \quad (1)$$

Where I_L is the limiting current (A), *n* is the number of electrons transferred in the reaction, F is Faraday's constant (C mol^{-1}), *A* is the geometrical surface area (cm^2), *D* is the diffusion coefficient of the reactant (cm^2 s^{-1}), ν is the kinematic viscosity of the solution (cm^2 s^{-1}), *C* is the concentration of the reactant (mol cm^{-3}), and ω is the rotational rate of the RDE (rad^{-1}). In this case, the reactant was O_2 in 0.5M H_2SO_4, and the value of C_{O2} is $1.15x10^{-5}$ mol cm^{-3} and D_{O2} is $1.61x10^{-6}$ cm^2 s^{-1} from literature.[8] The limiting current at 0.2 V vs. RHE was used as the Levich current (I_L).

NO Stripping:

The method of active site quantification was followed as described first by Malko et al.[9,10] In short, after an extensive electrochemical cleaning/wetting procedure, the ORR activity and the baseline capacitance of the un-poisoned catalyst were measured in O_2 and N_2 saturated 0.5 M acetate buffer respectively. The probe molecule was incorporated by soaking the electrode tip in a solution of 125 mM $NaNO_2$ for five minutes, where the NO_2^- molecule was reduced to NO after binding to the transition metal center of the macrocycle. The electrode was cleaned in DI

water and acetate buffer before transferring it to a solution of fresh electrolyte. The ORR activity of the poisoned catalyst was tested in O_2 saturated electrolyte at a scan rate of 5 mV/s before stripping the NO probe from 0.6 V to -0.3 V in a single scan at 10 mV/s. The NO probe molecule is assumed to be stripped via a proposed 5-electron reduction to NH_4^+, as shown below.

$$NO + 5e^- + 6H^+ \rightarrow NH_4^+ + H_2O \qquad (2)$$

The ORR activity of the catalyst was tested again to confirm complete recovery of the activity, indicating the total removal of the NO prob molecule.

The site density can then be determined as described below.

$$SD\ [sites\ nm^{-2}] = \frac{Q_{strip}[C\ g^{-1}] \times N_A[mol^{-1}]}{n_{strip}\ F[C\ mol^{-1}] \times SA[nm^2 g^{-1}]} \qquad (3)$$

Where *n* is the number of electrons used to reduce and strip off the probe molecule, which is assumed to be 5 electrons, as shown in the equation above. The stripping charge Q was determined by integrating the difference in the poisoned and unpoisoned CV in the region from 0.6 V to -0.3 V vs RHE. N_A and F are Avogadro's number and faradays constant, respectively, and the surface area *SA* is determined from the capacitance of the catalyst by,

$$SA[m^2] = \frac{C[F]}{C_S[F\ m^{-2}]} \qquad (4)$$

Where C is the double-layer capacitance of the material derived from cyclic voltammetry under argon and Cs is the specific capacitance of the material, assumed to be 204 mF m^{-2}, determined for carbon surfaces.[11,12] Here, we used the capacitance at 0.3V for the calculation of the surface area.

From the site density of the catalyst, the turnover frequency (TOF) can be calculated as seen in equation 5, and the kinetic current density at 0.8V vs. RHE, which allows for the calculation of the TOF from sites that are affected by the NO adsorption only giving a higher specificity for the active MN_4 sites.

$$TOF\ (@0.8V\ vsRHE)[s^{-1}] = \frac{\Delta i_k[Ag^{-1}]}{F\ [C\ mol^{-1}] \times MSD[mol\ g^{-1}]} \quad (5)$$

Where the molar site density (MSD) is the site density in moles d normalized to the mass, and the Δi_k is the difference between the kinetic current density at 0.8V of the poisoned and unpoisoned catalyst, with the kinetic current defined as,

$$i_k = \frac{i \times i_{lim}}{i_{lim} - i} \quad (6)$$

Where i was the current at 0.8V vs. RHE and i_{lim} was the limiting current in the RDE polarization curve.

Tables and Figures

Table S1. Fitting results of the in-temperature XAS Mn-K edge FT-EXAFS spectra shown in **Figure S2.**

**Sample**	**Independent Var.**	**# of Var.**	**χ^2**	**Red. χ^2**	**R-factor**	**E_0 (eV)**	**R (Å)**	**σ^2**	**N**
MnNC-BM-RT	7.0	4	1276	431	0.025	2.98(1.56)	2.17 (0.02)	0.010 (0.003)	3.9 (0.7)
MnNC-BM-1000	5.8	4	219	125	0.010	-3.55(2.22)	2.00 (0.03)	0.023 (0.005)	3.2 (0.7)

Table S2. Elemental analysis of MnNC-CVD-1100 catalyst and the nitrogen doped carbon (NC) by ICP-OES (Fe, Mn, and Zn), ion chromatography (Cl) and combustion analysis (C, H, and N).

	Fe (wt%)	Mn (wt%)	Zn (wt%)	Cl (wt%)	N (wt%)	C (wt%)	H (wt%)
MnNC-CVD-1100	122 ppm	1.01%	44 ppm	1017ppm	2.68%	88.67%	0.96%

NC	91 ppm	180 ppm	2.49%	594 ppm	5.19%	84.37%	1.04%

Table S3. Results active site density and turnover frequency as determined from NO stripping.

	SA (m^2 g^{-1})	SD (10^{16} sites m^{-2})	TOF @ 0.8V (e^{-1} $site^{-1}$) pH 5.2
Fe (CNRS)[10]	840	1.71	0.65
Fe (PAJ)[10]	593	0.42	7.23
Mn (This work)	726	0.29	3.1

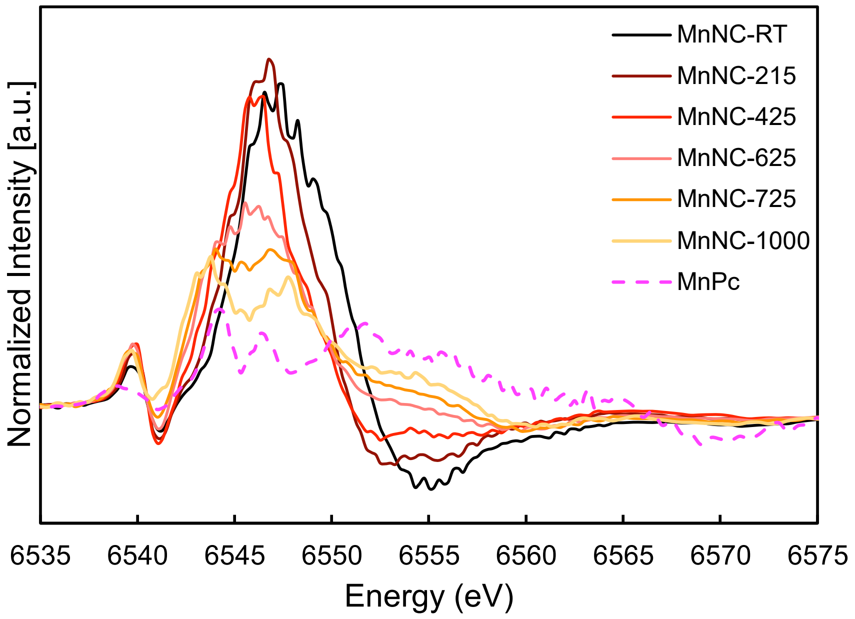

Figure S1. First derivative of XANES edge spectrum of in-temperature XAS during pyrolysis under inert gas held at various temperatrues during the heating process showing the emergance of the MnN4 fingerprint of MnPc at 6544 eV.

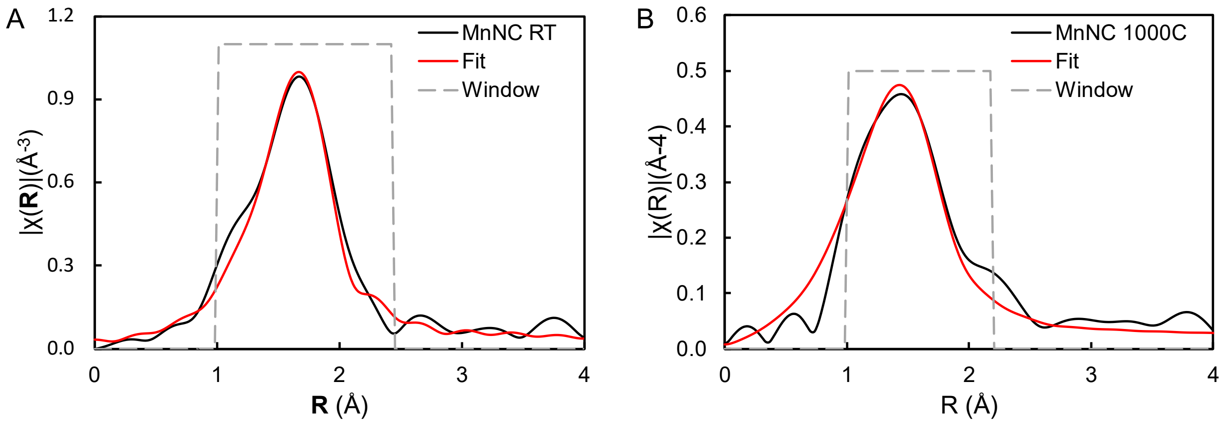

Figure S2. Mn K-edge FT-XAFS fitting of the in-temperature XAS data at (A) intial room temperature and (B) at 1000 °C. The fitting results are listed in **Table S1.**

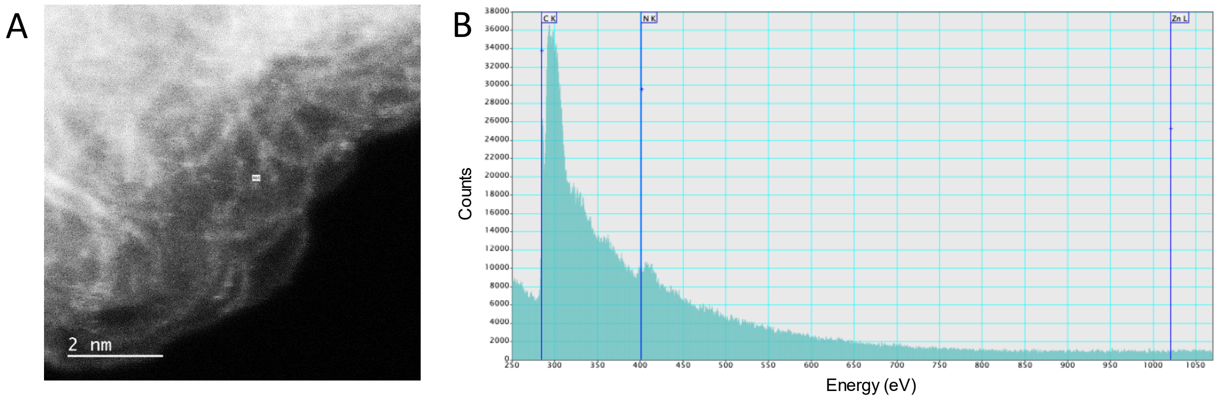

Figure S3. (A) High resolution ADF-STEM image of the NC and (B) EELS spectrum showing the C K-edge, N K-edge and Zn-L edge of the highlighted section surrounding a single metal atom.

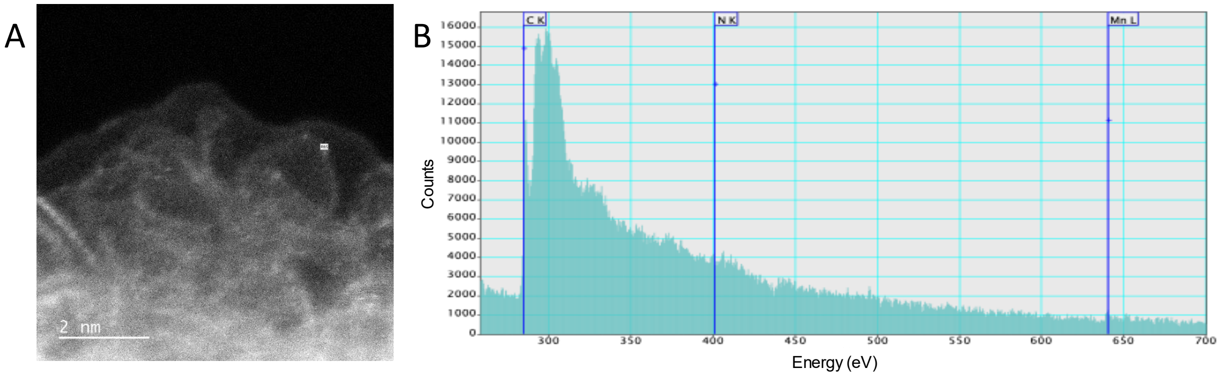

Figure S4. (A) High resolution ADF-STEM image of MnNC-CVD-1100 and (B) EELS spectrum showing the C K-edge, N K-edge and Mn-L edge of the highlighted section surrounding a single metal atom.

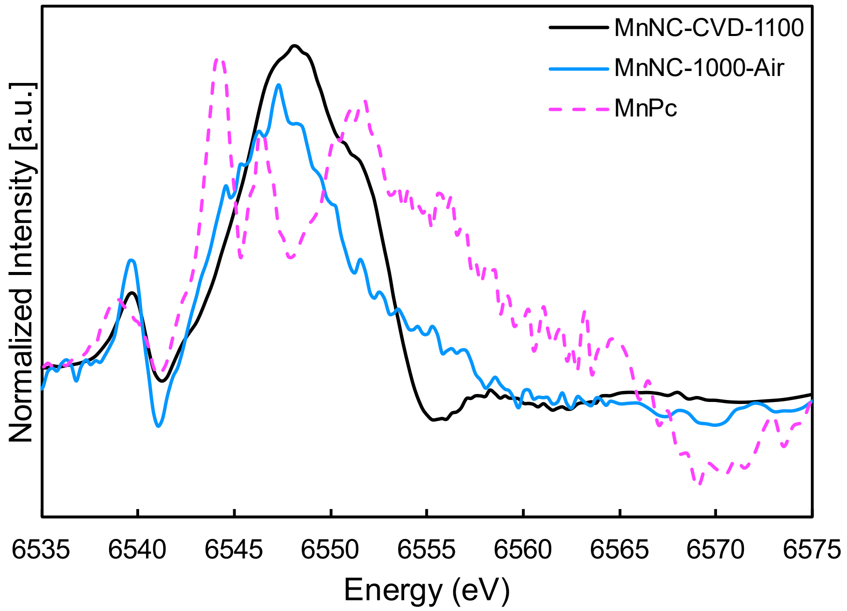

Figure S5. First derivative of XANES edge spectrum of the MnNC-CVD-1100 catalyst in comperision to the in-temperature MnNC 1000C after cooling and exposure to Air (MnNC-1000-Air) and the MnPc refence material.

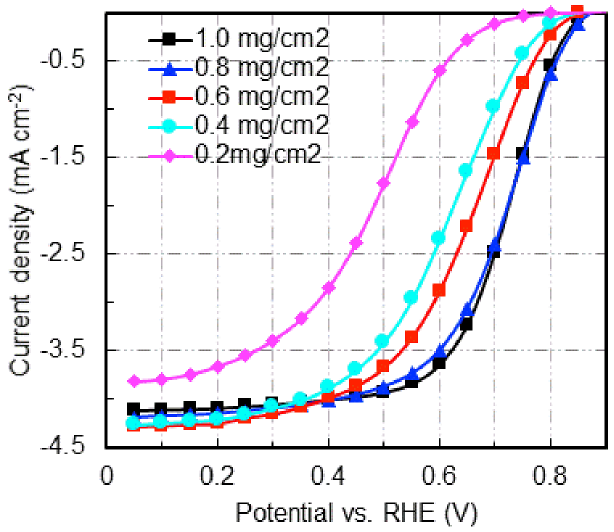

Figure S6. Loading dependance on ORR activity for the MnNC-CVD-1100 catalyst in O_2 purged 0.5M H_2SO_4 electrolyte at 900 RPM

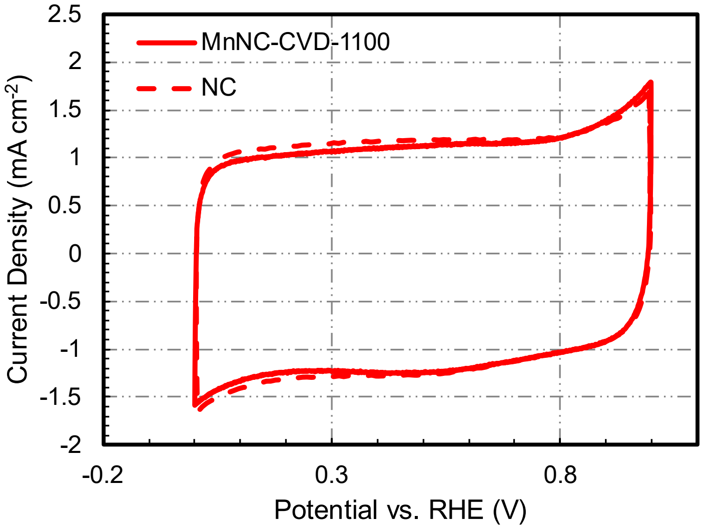

Figure S7. Cyclic voltammograms of the nitrogen doped carbon and the subsequent MnNC-CVD-1100 catalyst in N_2 saturated 0.5M H_2SO_4 at room temperature and a 10 mV s^{-1} scan rate.

References

(1) Kwon, H. T.; Jeong, H. K.; Lee, A. S.; An, H. S.; Lee, J. S. Heteroepitaxially Grown Zeolitic Imidazolate Framework Membranes with Unprecedented Propylene/Propane Separation Performances. *J. Am. Chem. Soc.* **2015**, *137* (38).

(2) Myers, D. J.; Kropf, A. J.; Yang, D. In Situ X-Ray Absorption Spectroscopy Characterization of Iron-Carbon-Nitrogen Oxygen Reduction Reaction Catalysts during Pyrolysis. In *in Meeting Abstracts (The Electrochemical Society).*; 2018; p 23322.

(3) Zelenay, P.; Myers, D. J.; Dinh, H.; More, K. L. ElectroCat (Electrocatalysis Consortium). In *U.S. Department of Energy's 2017 Annual Merit Review and Peer Evaluation Meeting*;

2017.

(4) Zelenay, P.; Myers, D. J. ElectroCat (Electrocatalysis Consortium). In *U.S. Department of Energy's 2018 Annual Merit Review and Peer Evaluation Meeting, Washington, DC.*; 2018.

(5) Li, J.; Jiao, L.; Wegener, E.; Richard, L. L.; Liu, E.; Zitolo, A.; Sougrati, M. T.; Mukerjee, S.; Zhao, Z.; Huang, Y.; et al. Evolution Pathway from Iron Compounds to Fe1(II)-N4 Sites through Gas-Phase Iron during Pyrolysis. *J. Am. Chem. Soc.* **2020**, *142* (3), 1417–1423.

(6) Newville, M. IFEFFIT : Interactive XAFS Analysis and FEFF Fitting. *J. Synchrotron Radiat.* **2001**, *8* (2), 322–324.

(7) Ankudinov, A. L.; Ravel, B.; Rehr, J. J.; Conradson, S. D. Real-Space Multiple-Scattering Calculation and Interpretation of x-Ray-Absorption near-Edge Structure. *Phys. Rev. B* **1998**, *58* (12), 7565.

(8) Ocampo, A. L.; Castellanos, R. H.; Sebastian, P. J. Kinetic Study of the Oxygen Reduction Reaction on Ruy(CO)n in Acid Medium with Different Concentrations of Methanol. *J. New Mater. Electrochem. Syst.* **2002**, *5* (3), 163–168.

(9) Malko, D.; Kucernak, A.; Lopes, T. In Situ Electrochemical Quantification of Active Sites in Fe-N/C Non-Precious Metal Catalysts. *Nat. Commun.* **2016**, *7*.

(10) Primbs, M.; Sun, Y.; Roy, A.; Malko, D.; Mehmood, A.; Sougrati, M.-T.; Blanchard, P.-Y.; Granozzi, G.; Kosmala, T.; Daniel, G.; et al. Establishing Reactivity Descriptors for Platinum Group Metal (PGM)-Free Fe-N-C Catalysts for PEM Fuel Cells †. **2020**.

(11) Zhou, Y. K.; He, B. L.; Zhou, W. J.; Huang, J.; Li, X. H.; Wu, B.; Li, H. L. Electrochemical Capacitance of Well-Coated Single-Walled Carbon Nanotube with

Polyaniline Composites. *Electrochim. Acta* **2004**, *49* (2), 257–262.

(12) Li, J.; Ghoshal, S.; Liang, W.; Sougrati, M. T.; Jaouen, F.; Halevi, B.; McKinney, S.; McCool, G.; Ma, C.; Yuan, X.; et al. Structural and Mechanistic Basis for the High Activity of Fe-N-C Catalysts toward Oxygen Reduction. *Energy Environ. Sci.* **2016**, *9* (7), 2418–2432.
